# The Role of Tumor Immune Microenvironment and Clinical Factors in Head and Neck Cancer Prognosis Among African American Men and Women

**DOI:** 10.3390/cancers18030481

**Published:** 2026-01-31

**Authors:** Shaynie Segal, Jianhong An, Matan Berkovsky, Geena Jung, Ashley Stone, Vicky Yau, Juan Lin, Richard V. Smith, Shanye Yin

**Affiliations:** 1 Albert Einstein College of Medicine, Bronx, NY 10461, USA; shaynie.segal@einsteinmed.edu (S.S.);; 2 Department of Pathology, Montefiore Medical Center, Bronx, NY 10461, USA; jianhong.an@einsteinmed.edu; 3 Department of Otolaryngology—Head and Neck Surgery, University of California San Francisco, San Francisco, CA 94143, USA; 4 Division of Oral and Maxillofacial Surgery, Mount Sinai Hospital, Icahn School of Medicine at Mount Sinai, New York, NY 10029, USA; 5 Department of Otorhinolaryngology—Head and Neck Surgery, Montefiore Medical Center, Bronx, NY 10467, USA; rsmith@montefiore.org

**Keywords:** microenvironment, HNSCC, racial disparities

## Abstract

**Background**: Head and Neck Squamous Cell Carcinoma (HNSCC) causes half a million deaths each year; therefore, it is essential to understand the factors that affect patient prognosis. Many studies fail to investigate the biological drivers behind survival disparities, especially sex-specific differences within racial groups. This study serves as a foundational project to begin elucidating biological differences in the tumor microenvironment between male and female African American HNSCC patients. **Methods**: A total of 111 patients who were diagnosed with HNSCC and identify as African American were grouped by sex. Analyses of socioeconomic status, co-morbidities, tumor characteristics, and treatment were conducted. Spatial transcriptomic analysis was performed on four randomly selected primary HNSCC tumor tissues. **Results**: No sex-based differences were observed in socioeconomic measures, treatments, tumor stage, follow-up, recurrence, or cause of death (all *p* > 0.15), though females had higher median income than males (*p* = 0.035). Comorbidity profiles were also largely comparable between males and females. Evaluating tumor microenvironments, we found that male tumors were dominated by malignant cells and fibroblasts, with limited adaptive immune infiltration. By contrast, female tumors displayed markedly higher proportions of immune cells, including T cells and B cells. Male tumors harbored sparse T cells, largely skewed toward exhausted phenotypes while female tumors displayed abundant T cell infiltration consistent with immunologically active tumor microenvironment. **Conclusions**: Clinical and demographic factors showed minimal sex-based differences among African American HNSCC patients, spatial transcriptomic profiling revealed strikingly distinct immune microenvironments by sex. These findings suggest that biological, rather than simply clinical, differences may drive survival disparities. This project serves as a novel and foundational study promoting the use of spatial transcriptomics to evaluate possible survival disparities within HNSCC populations to alleviate survival disparities.

## Simple Summary

Head and neck cancer affects hundreds of thousands of people worldwide each year, and survival outcomes are worse in African American patients. Prior studies have shown that African American women with this cancer live longer than African American men, but the reasons for this difference are not well understood. In this study, we examined clinical, social, and biological factors to better understand why this survival gap exists. We found that men and women had similar access to care, treatment patterns, and medical conditions, suggesting these factors do not fully explain the difference in outcomes. Using advanced tissue analysis, we observed that tumors from women showed stronger immune activity, while tumors from men had weaker immune responses. These findings suggest that biological differences in the tumor environment may contribute to survival disparities and could help guide more personalized cancer treatments in the future.

## 1. Introduction

Head and Neck Squamous Cell Carcinoma (HNSCC) is a malignancy that is responsible for 890,000 new cancer cases and 450,000 deaths globally per year [1]. Its high prevalence and significant mortality make it essential to understand factors that underpin patient prognoses. This is particularly important in addressing healthcare inequities, as minority and lower socio-economic communities experience both a higher incidence of HNSCC and poorer survival outcomes [1].

Historically, African American HNSCC patients have been found to have lower overall survival rates when compared to their White, Native American, Hispanic, and Asian counterparts [2]. Previous studies have identified some contributing factors, such as increased consumption of alcohol products and tobacco, prior viral exposure, and varying tumor factors at presentation [3]. Additional studies have found that clinical factors such as quality of hospital care [4] and a higher likelihood of surgical treatment refusal among African American patients also play a role in these disparities [5]. More recently, HPV exposure has been identified as another major negative prognostic factor for HNSCC [6], alongside other genetic markers that may be novel determinants for survival among African American HNSCC patients [7]. Yet, many studies fail to investigate any biological drivers behind these disparities, especially sex-specific differences within racial groups.

Therefore, the goal of this study is to serve as a preliminary and foundational study to address this critical gap in the understanding of within-race sex disparities in survival among African American patients with HNSCC. Jung et al. demonstrated a survival advantage for African American females compared to males, but despite this basic knowledge, the reasons for this disparity remain unclear [8]. While many studies have examined the reasons behind inter-race disparities, few have explored sex-based differences within the same race, leaving both the underlying clinically driven and tumor microenvironment-related mechanisms largely unexplored.

Tumor microenvironment remains an especially understudied dimension of HNSCC prognosis that may yield a deeper understanding of survival inequities among racial minorities. By integrating clinical and cellular data, this study seeks to begin clarifying the factors driving sex-based survival differences in African American patients. Addressing this gap could inform more personalized treatment regimens and more effective treatment approaches for African American HNSCC patients, ultimately taking the next step in improving survival outcomes and reducing inequities in this high-risk population.

### The Foundation of This Project

This study builds on previous work done by this group, which identified a significant survival difference between African American males and females with HNSCC (Jung et al., 2025) [8]. This study confirmed a sex-based survival disparity with females showing a survival advantage that persisted after adjusting for factors such as HPV status, comorbidities, tumor stage, and treatment type [8]. Additionally, African American females were more likely to begin treatment within 60 days of diagnosis compared to African American males (*p* = 0.033), yet the median Time to Treatment Initiation (TTI) for females was longer (40 days vs. 34 days for males) [8].

## 2. Methods

### 2.1. Study Population

This retrospective study included 111 patients (72% males, 28% females) identifying as Non-Hispanic African American who were diagnosed with HNSCC at Montefiore Medical Center from 1 February 2001 to 31 December 2020. This sex distribution is consistent with the established male predominance of HNSCC and reflects the underlying disease epidemiology rather than selective enrollment [9]. These are the same patients that we included in a prospective genomic study that did not represent all patients with head and neck cancer over this time frame [8]. However, all analyses reported in the present manuscript are novel. To be included, patients had to have biopsy-confirmed HNSCC affecting the lip or oral cavity, nasopharynx, oropharynx, larynx, or hypopharynx, and they needed to have completed treatment aimed at curing the cancer. Patients were excluded if they did not finish their primary cancer treatment, had an unknown origin of the cancer, or were diagnosed with non-aerodigestive HNSCC. This study was approved by the Albert Einstein Institutional Review Board (IRB #: 02-05-127E).

### 2.2. Data Collection

The following variables were collected from paper charts and electronic medical records and recorded into a secure REDCap and Excel spreadsheet database: patient demographics (sex, zip code, median household income, area deprivation index, Distressed Community Index Score (DCI), race, ethnicity, language, insurance, employment, marital status), co-morbidities (smoking status, pack-years, drinking status, ECOG performance status, diabetes, hypertension, CHF, COPD, emphysema, asthma, HIV, autoimmune disease, liver disease, nutritional deficiencies, thyroid disorders, anemia, history of malignancy, Charlston Co-Morbidity Index), diagnosis characteristics (age, location, HPV status), treatment variables, pathology on diagnosis and also possible recurrence, time to treatment initiation, follow up frequency with otolaryngologists, medical oncology, and radiation oncology, recurrence variables, survival status, and referrals.

### 2.3. Socioeconomic Measures

Area Deprivation Index (ADI) and employment rate were obtained from publicly available socioeconomic datasets linked to patient residential ZIP codes. To assess broader community-level socioeconomic conditions, we used the Distressed Communities Index (DCI), a composite ranking by ZIP code from 2018 to 2022 that incorporates seven indicators: unemployment rate, educational attainment, poverty rate, median income, change in the number of business establishments, job growth, and housing vacancy rate. Median income by ZIP code was extracted separately and analyzed using the Mann–Whitney U test to detect sex differences.

### 2.4. Treatment Classification

Index treatment type was categorized as: Surgery alone, Radiotherapy (RT) alone, Chemoradiotherapy (CRT), Surgery plus adjuvant RT or CRT. Post-operative treatment intensity for index tumors was defined as the number of additional interventions following the index treatment, including diagnostic procedures, surgery, radiation, chemotherapy, immunotherapy, or palliative care.

### 2.5. Follow-Up Measures

Follow-up attendance was defined as the proportion of scheduled post-treatment visits attended. Post-operative intervention referrals were counted as discrete additional referrals for care after initial treatment. Age at recurrence was recorded as the patient’s age at the time of recurrence diagnosis. Time to recurrence detection was measured from date of initial treatment to the date of documented recurrence. The number of follow-up visits before recurrence detection was counted for each patient. Treatment intensity for recurrent tumors was classified as the number of interventions using the categories of surgery, radiation, chemoradiation, surgery + radiation/chemoradiation, palliative care, or other. Cause of death was classified as: Recurrence, Complications from recurrence, Unrelated causes, Unknown, and Primary tumor.

### 2.6. Comorbidity Assessment

Comorbidities were abstracted from medical records and analyzed between sexes. All comorbidities evaluated are listed in Section 3.2. Table 2.

### 2.7. Time to Treatment Initiation (TTI)

TTI was defined as the number of days between histopathological diagnosis and initiation of first treatment, including RT, CRT, surgery alone, or surgery with adjuvant RT/CRT [10]. A 60-day threshold was used, based on prior literature demonstrating that initiating treatment more than 60 days after diagnosis is associated with worse survival outcomes [11].

### 2.8. Single-Cell Spatial Profiling

To explore potential biological mechanisms underlying the observed sex-based survival disparity identified in the main cohort of 111 African American HNSCC patients, a subset of four HPV-positive oropharyngeal squamous cell carcinoma (OPSCC) specimens (male, *n* = 2; female, *n* = 2) was selected for spatial multi-omics analysis. All four spatially profiled cases were between 44 and 57 years of age and had stage IV disease at diagnosis. All patients had a history of tobacco use and no major documented comorbidities. All patients received cisplatin-based neoadjuvant chemotherapy prior to surgery, and spatial multi-omics analysis was performed on surgically resected primary tumor specimens. These cases were selected to minimize biological heterogeneity, with uniform anatomic site (oropharynx), advanced stage, similar smoking exposure, absence of major comorbidities, and similar treatment exposure. Formalin-fixed paraffin-embedded (FFPE) tissue sections were examined using the G4X Spatial Sequencing System (Singular Genomics, San Diego, CA, USA), which allows parallel detection of RNA transcripts and protein markers in situ. In brief, 5-μm sections were mounted onto hydrogel-coated carriers, after which regions of interest (ROIs) were chosen and transferred to slides. Tissue samples then underwent deparaffinization, antigen retrieval, and flow cell assembly in accordance with the manufacturer’s instructions.

Multiplexed protein interrogation was achieved with an antibody-oligonucleotide conjugate panel, and padlock probe hybridization captured both RNA and protein targets. Subsequent signal amplification and sequencing were carried out on the G4X instrument. The transcriptome panel comprised 350 RNA probes, while a 15-marker antibody panel enabled simultaneous protein quantification. To visualize tissue morphology, nuclear and membrane dyes with H&E-like contrast were applied.

Spatial multi-omics datasets were processed within the scverse Python framework (Scanpy (v1.11.1) [12], Squidpy (v1.5.0) [13], with data stored and manipulated in AnnData objects). Cells and genes failing quality thresholds (low counts, outlier transcript levels, or abnormal nuclear morphology) were excluded. Data integration and clustering employed PCA, Harmony [14] for batch correction, and the Leiden community detection algorithm [15]. Cell identities were assigned using established RNA and protein markers. Finally, spatial indices such as Shannon entropy and local niche composition were quantified with Squidpy.

This analysis was conducted as an exploratory, proof-of-concept study due to the technical complexity and resource-intensive nature of the G4X spatial multi-omics platform. Specimens were selected to minimize biological heterogeneity and ensure tissue quality, based on uniform HPV status and anatomic site, adequate tumor cellularity, preserved tissue architecture, and immune infiltration as confirmed by hematoxylin and eosin evaluation and expert pathology review. Balanced sex representation was prioritized to enable exploratory assessment of sex-associated tumor microenvironment features.

### 2.9. Statistical Analysis

Chi-square tests were used to assess associations between categorical variables. Independent-sample *t*-tests were conducted to compare continuous variables between groups, and Mann–Whitney U tests were applied for non-normally distributed continuous data. All statistical tests were two-tailed, with a *p* value < 0.05 considered statistically significant. Analyses were performed using SPSS Statistics (version 31.0.0).

## 3. Results

Building on prior work by Jung et al. [8], which evaluated clinically relevant differences across racial groups, the present study reanalyzed the same dataset to focus specifically on African American males and females. As reported previously in Jung et al. (2025) [8], across the cohort, men and women were similar in language preference, age, marital and employment status, insurance type, area deprivation index, ECOG performance status, current tobacco and alcohol use, and HPV status (all *p* > 0.05). Women were less likely to have ever smoked (*p* = 0.036) or ever consumed alcohol (*p* = 0.004) [8]. Among African American patients, men and women showed no significant differences in index tumor stage, tumor location, or initial treatment approach (all *p* > 0.05). The only notable disparity was time to treatment initiation, with women more often starting treatment within 60 days (96.9% vs. 81.3%, *p* = 0.033) [8]. Recurrence rates trended lower in women but did not reach statistical significance [8]. Readers are encouraged to consult the original publication for the full version of the demographic table and its accompanying details.

### 3.1. Socioeconomic and Treatment Course Comparison

Analysis of additional socioeconomic indicators and treatment-related variables between African American males and females demonstrated minimal sex-based differences (Table 1). Median income was the only socioeconomic measure that differed significantly between groups (*p* = 0.035). In contrast, the Distressed Communities Index (DCI), a composite measure of community-level socioeconomic deprivation, did not differ between males and females (*p* = 0.515). Full distributions for both median income and DCI are available in Supplemental Tables S1 and S2. Additional *p* values and distributions can be found in Supplemental Tables S3–S5. 

Regarding treatment characteristics, males and females demonstrated comparable patterns across the examined variables. The number of initial post-operative interventions for the index tumor (*p* = 0.972) and the number of post-operative referrals (*p* = 0.764) did not differ by sex. Index tumor–related measures were also similar, including final tumor stage (*p* = 0.454). Recurrence-related measures showed the same pattern of consistency between groups, with no significant differences in the number of recurrent tumor locations (*p* = 0.201), the presence of multiple recurrent sites (*p* = 0.192), or the final recurrent tumor stage (*p* = 0.645). Time to recurrence detection (*p* = 0.154), the number of follow-up visits before recurrence (*p* = 0.633), and overall follow-up visit attendance (*p* = 0.764) likewise showed no sex-based variation. Age at recurrence did not differ between groups (*p* = 0.852), nor did treatment intensity for recurrent tumors (*p* = 0.466). Finally, cause-of-death distributions remained similar across sexes (*p* = 0.414).

### 3.2. Co-Morbidity Comparison

Comparison of comorbid conditions between African American males and females revealed that most comorbidities were similarly distributed across groups (Table 2). Autoimmune diseases were the only comorbidity that differed significantly by sex (*p* = 0.025), with a higher prevalence among females. All other comorbidities, including hypertension (*p* = 0.08), uncomplicated diabetes (*p* = 0.817), complicated diabetes (*p* = 0.863), overall diabetes status (*p* = 0.757), congestive heart failure (*p* = 0.863), chronic obstructive pulmonary disease (*p* = 0.702), emphysema (*p* = 0.471), asthma (*p* = 0.622), HIV (*p* = 0.904), liver disease (*p* = 0.626), nutritional deficiencies (*p* = 0.870), thyroid disease (*p* = 0.483), and anemia (*p* = 0.689), did not differ significantly between sexes.

Given the similarity between groups across many clinical measures, subsequent analyses focused on tumor microenvironment profiles to identify potential biological contributors to the observed survival disparity.

### 3.3. Sex-Specific Immune Microenvironments in Black Head and Neck Squamous Cell Carcinoma (HNSCC) Patients

**Figure 1:**
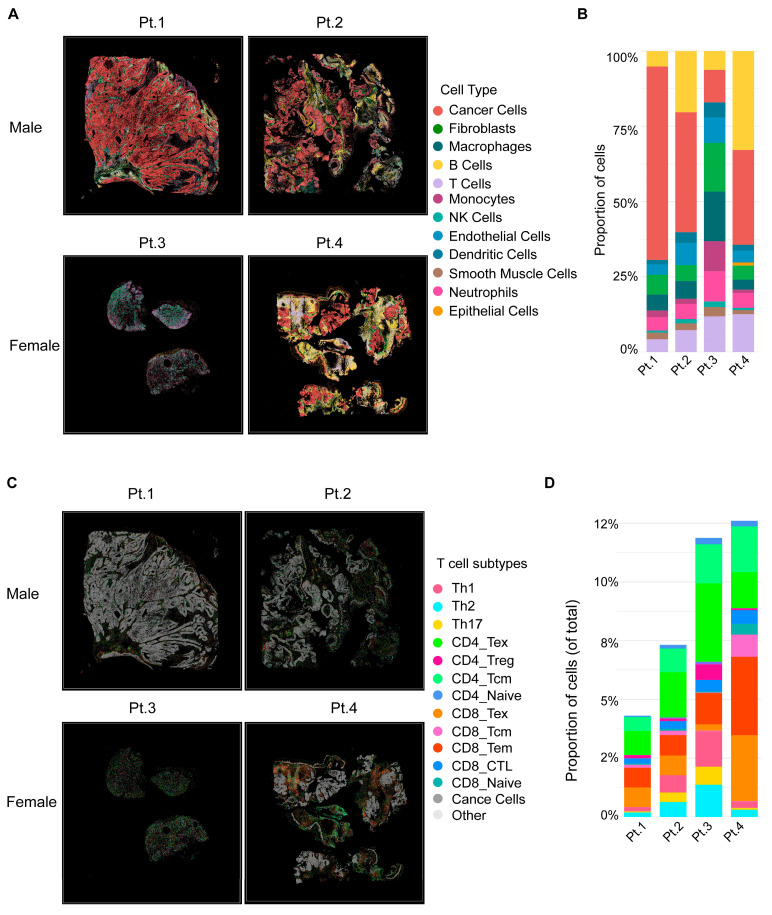
Spatial transcriptomic analysis of Black male and female HNSCC tumors. (**A**) Two male (Pt.1, Pt.2) and two female (Pt.3, Pt.4) patients with primary oropharyngeal tumors. Male tumors are dominated by malignant cells with limited immune infiltration, while female tumors show higher immune content. (**B**) Stacked bar plots quantifying the proportion of major cell types. Male tumors exhibit stromal and myeloid dominance, whereas female tumors display increased T and B cells. (**C**) Spatial maps of T cell subpopulations across the same tumors. (**D**) Quantification of T cell subsets. Male tumors are enriched for exhausted and regulatory T cells, while female tumors harbor abundant and diverse effector subsets, including CD8_CTL and CD8_Tem.

Spatial transcriptomic analyses were performed on four representative primary oropharyngeal HNSCC specimens from Black patients, including two males (Pt.1, Pt.2) and two females (Pt.3, Pt.4). At the global level (Figure 1A,B), male tumors were dominated by malignant cells, with limited adaptive immune infiltration. The immune cells that were present were largely skewed toward myeloid populations, including macrophages and monocytes, consistent with a immunosuppressive niche. By contrast, female tumors displayed markedly higher proportions of immune cells, including T cells and B cells. This cellular landscape reflected an immune-inflamed microenvironment enriched for adaptive immune subsets.

Further dissection of T cell subpopulations (Figure 1C,D) revealed a profound immune divergence between male and female patients. Male tumors harbored sparse T cells, largely skewed toward exhausted phenotypes (CD4/8_Tex) and regulatory T cells (CD4_Treg), with minimal effector subsets. In contrast, female tumors displayed abundant T cell infiltration with diverse functional lineages, including cytotoxic CD8_CTL, CD8_Tem, suggesting a more immunologically active tumor microenvironment.

Together, these results demonstrate that male tumors are characterized by stromal and myeloid dominance with limited, exhausted T cell infiltration, while female tumors exhibit robust lymphoid infiltration with functional effector T cell subsets. These spatially resolved immune phenotypes suggest a potential biological contributor for the clinical observation that Black male HNSCC patients have worse outcomes, whereas Black female patients tend to experience more favorable prognosis.

## 4. Discussion

Previous research by Jung et al. (2025) [8] demonstrated a significant survival difference between African American males and females with HNSCC, independent of HPV status, comorbidities, tumor stage, and treatment type. This study extends those findings by reanalyzing the same dataset with a focus on intra-racial sex differences among African American patients. Uniquely, it integrates spatial transcriptomic profiling to characterize, for the first time, sex-specific features of the tumor microenvironment in African American HNSCC, providing new insights into the biological mechanisms that may drive the observed survival disparities. The results suggest a potential cellular basis for these sex-based differences in survival, as African American females had an increased immunologic reaction to the tumor cells versus their male counterparts paving the way for these differences to be further explored. Although the sample size is modest, these findings establish an important foundation for future studies aimed at elucidating the molecular determinants of survivability differences between African American men and women with HNSCC. Although many prior studies explored the cellular basis of disease prognosis and survival by sex or between races, these findings are the first to evaluate and potentially identify such tumor microenvironment differences specifically between African American males and females.

Prior genomic studies have primarily focused on racial differences in HNSCC biology, which have provided an important context for understanding disparities in disease behavior. For instance, Nogueira et al., found that MSH2 GG genotype was more prevalent among White patients than those of other races, and that patients with reduced MSH2 were more prone to developing severe laryngeal squamous cell carcinoma [16]. Chaudhary et al. further demonstrated that African American patients with HNSCC have poorer prognoses than White patients, even after adjusting for many socioeconomic factors. Their analyses demonstrated that African American patients had increased mutation rates in key genes for tumor growth such as *p53* and *HRAS*, and also revealed important tumor microenvironment differences which included reduced infiltration of effector immune cells compared to their white counterparts [17]. This study established a foundation for comparing tumor microenvironments for HNSCC between races. Guerrero-Preston further elucidated the widespread differences in genomic expression between black and white HNSCC patients to provide a greater understanding of racial survival disparities [18]. They found elevated mutation rates in black patients (*p53*, *JAK3*, *MET*, and *NOTCH1*), with methylation differences varying by tumor site within the head and neck [14].

Together, these studies have elucidated potential molecular and immunologic underpinnings of racial disparities in HNSCC outcomes. However, none have examined whether similar mechanisms may underlie sex-based differences within the African American population. The present findings expand upon this literature by emphasizing potential distinct immunologic and molecular differences between African American males and females, a comparison largely overlooked in Otorhinolaryngological research.

As there were no sex-based differences in ADI, employment, DCI, treatment, interventions, tumor stage, follow-up, recurrence, or cause of death, these results further emphasize the possibility for biological contributions to survival disparities. Co-morbidities were also largely comparable between males and females within our population. Autoimmune diseases were more prevalent among females, a finding consistent with the known higher incidence of autoimmune conditions in women [19]. Notably, autoimmune disease is often associated with heightened immune activation, which may plausibly align with the more immune-inflamed tumor microenvironment observed in female tumors in this study. Additionally, it is important to note that hypertension demonstrated a non-significant trend toward higher prevalence among females; however, given its high baseline prevalence in head and neck cancer populations and its lack of a known association with enhanced antitumor immune responses, this finding is unlikely to meaningfully contribute to the observed sex-based differences in tumor immune microenvironment or survival. Overall, the limited and expected nature of these comorbidity differences further supports the conclusion that biological factors, rather than clinical comorbidity burden, may play a central role in driving sex-based survival disparities [20,21].

Interestingly, females within the population had a higher median income than males which could contribute to improved survival by enabling greater access to timely and comprehensive cancer care. This finding was unexpected and raises the possibility that socioeconomic differences may contribute to the survival disparities that we are observing, at least in part. However, because income was assessed at the ZIP code level, it may not accurately reflect individual socioeconomic circumstances. Importantly, broader measures of community-level deprivation, including the Area Deprivation Index and the Distressed Communities Index, did not differ between sexes, suggesting that men and women in this cohort were drawn from similarly disadvantaged communities. Thus, while females are experiencing a higher median income, this factor may only contribute modestly and is unlikely to explain the disparities observed within our population. Additionally, time to treatment initiation (TTI) was evaluated in Jung et al. and could additionally contribute to the survival disparities within our population [8]. Of the patients who began treatment within 60 days, females had a longer TTI than their male counterparts, but women were overall more likely to begin treatment within the 60 days [8]. It is important to acknowledge that this could be one of the contributing factors to the survival disparities observed within the African American population. Only patients who finished their primary cancer treatment were included, so nonadherence was not a confounding factor within our study. Together, these clinical findings highlight that despite minimal differences in demographic, socioeconomic, and treatment-related factors between African American males and females, survival disparities persist. This absence of clear clinical explanations underscores the likelihood that biological mechanisms, such as sex-specific tumor immune microenvironments, are central to driving survival outcomes.

To investigate this possibility, subsequent analyses focused on tumor immune microenvironments via spatial transcriptomics. These spatial transcriptomic findings provide possible mechanistic insights into the observed clinical disparity that Black male HNSCC patients experience worse survival compared with their female counterparts. The predominance of fibroblasts and immune-depleted niches in male tumors is consistent with an immune-excluded phenotype, where a dense stromal barrier restricts immune cell infiltration. Even when T cells were present, they exhibited an exhausted or immunosuppressive profile, limiting antitumor immunity. By contrast, female tumors demonstrated an immune-inflamed phenotype, characterized by higher immune infiltration and functional T cell diversity, including effector and helper subsets critical for orchestrating anti-tumor responses. This active immune contexture may underlie the more favorable prognosis observed in female patients.

Clinically, these findings suggest that Black male HNSCC patients may respond poorly to immune checkpoint blockade (ICB) alone, as exhausted and excluded T cells are less likely to be reactivated by PD-1/PD-L1 inhibition. Combination strategies targeting the fibrotic stroma or enhancing T cell priming may be required. In contrast, female patients may derive greater benefit from ICB monotherapy or immune-activating regimens, reflecting a more permissive tumor immune landscape. Overall, these results reveal possible sex-specific immune evasion strategies in HNSCC, emphasizing the need to integrate sex and ancestry as critical biological variables in the design of future immunotherapy-based treatments. Ultimately, it is also important to acknowledge that the survival disparities observed are likely multifaceted with income, time to treatment, and biological factors intertwined in affecting the survivability of patients.

This study is not without limitations. As a retrospective, single-institution analysis, the applicability of our findings to broader populations may be constrained. Furthermore, since these patients were originally enrolled in a prospective genomic study, the cohort did not involve all individuals with head and neck cancer during this time period. Additionally, only a small subset of patients underwent spatial transcriptomics. The modest sample size positions this work as a foundational step, intended to stimulate further research into the tumor microenvironment underpinning survival disparities among African American males and females. Future research should expand this approach to larger, multi-institutional cohorts and incorporate additional spatial transcriptomic profiling to validate and extend these findings. This will be essential to determining whether our observations can ultimately inform immunotherapy strategies and change clinical practice.

An additional limitation is the imbalance in sex distribution within the cohort, with males comprising 72% and females 28% of the study population. This disparity reflects the known epidemiology of head and neck squamous cell carcinoma, which disproportionately affects men; however, it may reduce statistical power for detecting sex-specific clinical differences and limits the precision of estimates among female patients [9]. Importantly, the primary objective of this study was not to compare incidence or prevalence by sex, but rather to evaluate whether differences in clinical characteristics or tumor immune microenvironment could plausibly contribute to previously observed sex-based survival disparities within an African American population. Despite the smaller female sample size, clinical, socioeconomic, and treatment-related variables remained largely comparable between sexes, suggesting that the observed immune microenvironment differences are unlikely to be driven solely by sampling imbalance. Nevertheless, these findings should be interpreted as hypothesis-generating, and future studies with larger and more balanced cohorts are needed to validate sex-specific biological differences in African American HNSCC.

Overall, this study provides valuable insight into a Bronx-based population that remains markedly understudied despite facing disproportionate health burdens. By highlighting unique disparities within this population, our findings underscore the need for broader, collaborative efforts to ensure a more comprehensive understanding of HNSCC outcomes across diverse populations and stimulate further research into using spatial transcriptomics to identify individual differences in tumor microenvironment with HNSCC.

## 5. Conclusions

This study identifies the possibility of a distinct sex-specific immune landscape in the sampled African American patients with HNSCC that may underlie the survival disparities previously described. Tumors from males more frequently demonstrated features of immune exclusion and exhaustion, potentially driving poorer outcomes. In contrast, tumors from females exhibited a more robust and functional lymphoid infiltration, which may help explain their comparatively improved survival.

These findings highlight the importance of elucidating the biological foundation of disease to develop a deeper understanding of survival inequities among racial minorities. By identifying the potential cellular and immunologic drivers of these disparities, there is an opportunity to develop personalized treatment strategies that address the unique tumor microenvironments of African American males and females. This project serves as a novel and foundational study promoting the use of spatial transcriptomics to evaluate survival disparities within HNSCC populations. Together, these insights lay the groundwork for biologically informed interventions aimed at reducing sex-based survival disparities in African American patients with HNSCC.

## Figures and Tables

**Table 1:** *p*-Values for Socioeconomic and Treatment Course Comparison Between African American Males and Females.

		Male (*n*, %)	Female (*n*, %)	*p*-Value
Treatment Intensity for Post-Operative Interventions of Index Tumor	1	5 (6.4)	2 (6.3)	0.972
	2	19 (24.4)	12 (37.5)	
	3	38 (48.7)	15 (46.9)	
	4	16 (20.5)	3 (9.4)	
Number of Post-Operative Referrals	0	48 (60.8)	16 (50.0)	0.764
	1	20 (25.3)	13 (40.6)	
	2	6 (7.6)	1 (3.1)	
	3	3 (3.8)	1 (3.1)	
	4	2 (2.5)	0 (0)	
	5	0 (0)	1 (3.1)	
Final Index Tumor Stage	I	7 (8.9)	4 (12.5)	0.454
	II	8 (10.1)	7 (21.9)	
	III	14 (17.7)	6 (18.8)	
	IVa	45 (57.0)	14 (43.8)	
	IVb	4 (5.1)	1 (3.1)	
	IVc	1 (1.3)	0 (0)	
Follow-up Visit Attendance (Percent of attended scheduled visits rounded)	20%	1 (1.3)	0 (0)	0.764
	40%	1 (1.3)	0 (0)	
	50%	3 (3.8)	2 (6.1)	
	60%	4 (5.1)	3 (9.1)	
	70%	1 (1.3)	1 (3.0)	
	80%	5 (6.3)	3 (9.1)	
	90%	8 (10.1)	1 (3.0)	
	100%	39 (49.4)	15 (45.5)	
Treatment Intensity for Post-Operative Interventions of Recurrent Tumors	0	1 (3.6)	0 (0)	0.466
	1	23 (82.1)	4 (66.7)	
	2	3 (10.7)	2 (33.3)	
	3	(3.6)	0 (0)	
Number of Recurrent Tumor Locations	1	20 (76.9)	6 (100.0)	0.201
	2	4 (15.4)	0 (0)	
	3	2 (7.7)	0 (0)	
Multiple Recurrent Tumor Locations (Y/N)	No, one location	20 (76.9)	6 (100.0)	0.192
	Yes, multiple locations	6 (23.1)	0 (0.0)	
Recurrent Tumor Final Stage	I	0 (0.0)	0 (0.0)	0.645
	II	2 (16.7)	1 (33.3)	
	III	1 (8.3)	1 (33.3)	
	IVa	5 (41.7)	1 (33.3)	
	IVb	1 (8.3)	0 (0.0)	
	IVc	3 (25.0)	0 (0.0)	
Cause of Death	Recurrent Tumor	18 (34.6)	3 (18.8)	0.414
	Complications from Recurrence	0 (0)	0 (0)	
	Other/Unrelated to Recurrence	27 (51.9)	9 (56.3)	
	Unknown	6 (11.5)	4 (25.0)	
	Primary Tumor	1 (1.9)	0 (0)	

**Table 2:** *p*-Values for Co-Morbidity Comparison Between African American Males and Females.

Variable	Male (*n*, %)	Female (*n*, %)	*p*-Value
Uncomplicated Diabetes	11 (13.9)	5 (15.6)	0.817
Complicated Diabetes	3 (3.8)	1 (3.1)	0.863
Diabetes (Y/N)	18 (22.8)	5 (15.2)	0.757
Hypertension	41 (51.9)	24 (72.7)	0.08
Congestive Heart Failure	3 (3.8)	1 (3.1)	0.863
COPD	20 (25.3)	7 (21.9)	0.702
Emphysema	3 (3.8)	0 (0)	0.471
Asthma	9 (11.4)	6 (18.2)	0.622
HIV	8 (10.1)	3 (9.4)	0.904
Autoimmune Diseases	0 (0)	2 (6.3)	0.025 *
Liver Disease	10 (12.7)	3 (9.4)	0.626
Nutritional Deficiencies	10 (12.7)	4 (12.1)	0.870
Thyroid Disease	5 (6.3)	4 (12.1)	0.483
Anemia	20 (25.3)	10 (30.3)	0.689

* Indicates statistical significance (*p* < 0.05).

## Data Availability

The data presented in this study are available on reasonable request from the corresponding author. The data are not publicly available due to ethical restrictions and patient privacy considerations.

## Supplementary Materials

The following supporting information can be downloaded at: https://www.mdpi.com/article/10.3390/cancers18030481/s1, Table S1: *p*-Values for DCI Comparison Between African American Males and Females; Table S2: *p*-Values for Income Comparison Between African American Males and Females; Table S3: *p*-Values for Age at Recurrence Comparison Between African American Males and Females; Table S4: *p*-Values for Time to Recurrence Detection (months) Comparison Between African American Males and Females; Table S5: *p*-Values for Number of Follow-Up Visits Before Recurrence Detected Comparison Between African American Males and Females.
