# Supplementary material for: The Role of Tumor Immune Microenvironment and Clinical Factors in Head and Neck Cancer Prognosis Among African American Men and Women
Source: Cancers (Basel). 2026 Jan 31;18(3):481. doi: 10.3390/cancers18030481 (PMC12896851; doi:10.3390/cancers18030481)
Supplement: Supplementary file 1 [file cancers-18-00481-s001.zip › cancers-4094636-supplementary.pdf]

Supplemental Tables

Supplemental Table S1. P-Values for DCI Comparison Between African American Males and Females

Supplemental Table S2. P-Values for Income Comparison Between African American Males and Females

Supplemental Table S3. P-Values for Age at Recurrence Comparison Between African American Males and Females

Supplemental Table S4. P-Values for Time to Recurrence Detection (months) Comparison Between African American Males and Females

Supplemental Table S5. P-Values for Number of Follow-Up Visits Before Recurrence Detected Comparison Between African American Males and Females

Supplemental Table S1. P-Values for DCI Comparison Between African American Males and Females.

		Sex			
		Male		Female	
		N	%	N	%
**DCI (p=0.515)**	.00	1	1.3%	0	0.0%
	24.80	0	0.0%	1	3.0%
	39.00	1	1.3%	0	0.0%
	42.50	0	0.0%	1	3.0%
	57.30	1	1.3%	0	0.0%
	58.30	1	1.3%	0	0.0%
	58.90	1	1.3%	0	0.0%
	60.50	1	1.3%	0	0.0%
	62.00	0	0.0%	1	3.0%
	66.20	1	1.3%	0	0.0%
	70.70	2	2.6%	4	12.1%
	72.60	1	1.3%	0	0.0%
	73.20	1	1.3%	0	0.0%
	75.20	1	1.3%	0	0.0%
	76.40	1	1.3%	0	0.0%
	76.90	3	3.8%	2	6.1%
	79.60	5	6.4%	0	0.0%
	80.70	1	1.3%	0	0.0%
	81.70	2	2.6%	0	0.0%
	81.90	1	1.3%	0	0.0%
	82.10	1	1.3%	1	3.0%
	83.10	2	2.6%	2	6.1%
	83.50	1	1.3%	0	0.0%
	84.00	5	6.4%	5	15.2%
	84.50	0	0.0%	1	3.0%
	85.30	1	1.3%	0	0.0%
	85.40	11	14.1%	5	15.2%
	86.30	3	3.8%	1	3.0%
	87.50	7	9.0%	2	6.1%
	89.00	1	1.3%	0	0.0%
	90.10	10	12.8%	1	3.0%
	90.20	1	1.3%	1	3.0%
	90.40	3	3.8%	2	6.1%
	90.70	2	2.6%	1	3.0%
	92.20	3	3.8%	2	6.1%
	92.70	2	2.6%	0	0.0%

Supplemental Table S2. P-Values for Income Comparison Between African American Males and Females.

		Sex			
		Male		Female	
		N	%	N	%
**Income (p=0.035)**	10340.00	0	0.0%	1	3.0%
	30741.00	1	1.3%	0	0.0%
	31166.00	2	2.6%	0	0.0%
	31504.00	1	1.3%	2	6.1%
	33877.00	8	10.3%	3	9.1%
	33945.00	2	2.6%	2	6.1%
	34860.00	2	2.6%	3	9.1%
	36631.00	1	1.3%	0	0.0%
	36730.00	3	3.8%	1	3.0%
	38066.00	7	9.0%	1	3.0%
	38768.00	1	1.3%	1	3.0%
	40138.00	3	3.8%	2	6.1%
	42639.00	8	10.3%	1	3.0%
	45616.00	0	0.0%	1	3.0%
	46730.00	0	0.0%	1	3.0%
	48212.00	1	1.3%	1	3.0%
	48472.00	2	2.6%	2	6.1%
	55553.00	1	1.3%	0	0.0%
	56327.00	7	9.0%	1	3.0%
	57180.00	1	1.3%	0	0.0%
	58435.00	1	1.3%	0	0.0%
	59032.00	5	6.4%	0	0.0%
	61087.00	2	2.6%	0	0.0%
	61414.00	0	0.0%	1	3.0%
	62918.00	7	9.0%	2	6.1%
	63048.00	1	1.3%	0	0.0%
	65943.00	1	1.3%	0	0.0%
	66546.00	1	1.3%	0	0.0%
	70107.00	0	0.0%	1	3.0%
	70415.00	4	5.1%	2	6.1%
	73632.00	1	1.3%	0	0.0%
	75336.00	0	0.0%	1	3.0%
	78473.00	0	0.0%	1	3.0%
	80245.00	0	0.0%	1	3.0%
	88341.00	1	1.3%	0	0.0%
	89091.00	1	1.3%	0	0.0%
	96368.00	1	1.3%	0	0.0%
	101853.00	1	1.3%	0	0.0%
	211696.00	0	0.0%	1	3.0%

Supplemental Table S3. P-Values for Age at Recurrence Comparison Between African American Males and Females.

		Sex			
		Male		Female	
		N	%	N	%
**Age at Recurrence (p=0.852)**	20.00	1	3.6%	0	0.0%
	33.00	1	3.6%	0	0.0%
	39.00	1	3.6%	0	0.0%
	48.00	1	3.6%	0	0.0%
	50.00	0	0.0%	1	16.7%
	52.00	1	3.6%	0	0.0%
	54.00	1	3.6%	0	0.0%
	56.00	1	3.6%	0	0.0%
	58.00	1	3.6%	0	0.0%
	61.00	2	7.1%	0	0.0%
	62.00	0	0.0%	1	16.7%
	63.00	2	7.1%	0	0.0%
	64.00	2	7.1%	0	0.0%
	68.00	1	3.6%	1	16.7%
	69.00	2	7.1%	0	0.0%
	73.00	1	3.6%	1	16.7%
	74.00	3	10.7%	0	0.0%
	76.00	1	3.6%	0	0.0%
	77.00	2	7.1%	0	0.0%
	78.00	1	3.6%	1	16.7%
	80.00	1	3.6%	0	0.0%
	81.00	1	3.6%	0	0.0%
	82.00	1	3.6%	0	0.0%
	92.00	0	0.0%	1	16.7%

Supplemental Table S4. P-Values for Time to Recurrence Detection (months) Comparison Between African American Males and Females.

		Sex			
		Male		Female	
		N	%	N	%
**Time to Recurrence Detection (months) (p=0.154)**	.00	1	3.6%	0	0.0%
	2.00	1	3.6%	0	0.0%
	3.00	1	3.6%	0	0.0%
	4.00	3	10.7%	0	0.0%
	6.00	1	3.6%	0	0.0%
	7.00	1	3.6%	0	0.0%
	8.00	0	0.0%	1	16.7%
	9.00	2	7.1%	0	0.0%
	10.00	3	10.7%	0	0.0%
	11.00	4	14.3%	0	0.0%
	12.00	1	3.6%	0	0.0%
	13.00	1	3.6%	1	16.7%
	14.00	0	0.0%	1	16.7%
	15.00	1	3.6%	0	0.0%
	21.00	0	0.0%	1	16.7%
	22.00	1	3.6%	0	0.0%
	23.00	1	3.6%	0	0.0%
	25.00	1	3.6%	0	0.0%
	26.00	1	3.6%	0	0.0%
	28.00	0	0.0%	1	16.7%
	36.00	1	3.6%	0	0.0%
	37.00	1	3.6%	0	0.0%
	83.00	0	0.0%	1	16.7%
	100.00	1	3.6%	0	0.0%
	119.00	1	3.6%	0	0.0%

Supplemental Table S5. P-Values for Number of Follow-Up Visits Before Recurrence Detected Comparison Between African American Males and Females.

		Sex			
		Male		Female	
		N	%	N	%
**Number of Follow-up Visits Before Recurrence Dete cted (p=0.633)**	.00	5	17.9%	1	16.7%
	1.00	5	17.9%	0	0.0%
	2.00	4	14.3%	1	16.7%
	3.00	2	7.1%	1	16.7%
	5.00	1	3.6%	1	16.7%
	6.00	1	3.6%	0	0.0%
	7.00	1	3.6%	0	0.0%
	9.00	3	10.7%	0	0.0%
	10.00	2	7.1%	1	16.7%
	12.00	1	3.6%	0	0.0%
	18.00	1	3.6%	1	16.7%
	25.00	1	3.6%	0	0.0%
	36.00	1	3.6%	0	0.0%
